# Updates on antifungal pharmacotherapy in elasmobranchs: pharmacokinetics of 4 mg/kg voriconazole after IM and IV administration in undulate skates (*Raja undulata*) maintained under human care

**DOI:** 10.3389/fvets.2024.1376851

**Published:** 2024-06-06

**Authors:** Daniela Cañizares-Cooz, Carlos Rojo-Solís, Sonia Rubio-Langre, Daniel García-Párraga, Teresa Encinas, Pablo Morón-Elorza

**Affiliations:** ^1^Department of Pharmacology and Toxicology, Faculty of Veterinary Medicine, Complutense University of Madrid, Madrid, Spain; ^2^Veterinary Services, Oceanogràfic, Ciudad de las Artes y las Ciencias. C/ Eduardo Primo Yúfera (Científic) 1B, Valencia, Spain; ^3^Research Department, Fundación Oceanogràfic de la Comunitat Valenciana, Oceanogràfic, C/ Eduardo Primo Yúfera (Científic) 1B, Valencia, Spain

**Keywords:** antimycotic, shark, ray, chondrichthyans, pharmacology, *Fusarium*, half-life, azoles

## Abstract

**Introduction:**

Fungal diseases are frequently associated with elevated mortality rates in elasmobranchs. Currently, there is a notable absence of scientifically validated therapeutic medications that can ensure both effectiveness and safety when administered to this group of animals. The empirical prescription of azole antifungal agents, particularly voriconazole, has been posited as a potentially efficacious treatment approach for addressing most common mycoses in sharks and rays. However, there are still no published pharmacokinetic studies supporting its use in elasmobranchs and there is a lack of scientific base for its utilization in elasmobranchs.

**Methods:**

For this study, voriconazole was administered intravenously (IV) and intramuscularly (IM), at a single dose of 4 mg/kg to six adult undulate skates (*Raja undulata*). A washout period of 8 weeks was left between each route of administration. Blood samples were collected both before and at ten predetermined intervals after each dosing (0.25, 0.5, 1, 1.5, 2, 4, 8, 12, 24, and 36 h after drug administration). Plasma concentrations were quantified using a validated high-performance liquid chromatography method, and pharmacokinetic (PK) data was analyzed through non-compartmental methods.

**Results:**

The mean extrapolated concentration at 0 h (C_0_) after IV administration was 27.19 ± 7.15 μg/mL and the mean peak plasma concentrations (C_max_) ± SEM after IM administration resulted 2.98 ± 0.28 μg/mL at a mean time to maximum concentration (T _max_) of 1.33 ± 0.17 h. Terminal half-lives were calculated and resulted 11.18 ± 1.32 h for IV injections and 9.59 ± 1.38 h for IM injections. The area under the curve extrapolated to infinity was determined as 58.14 ± 2.79 h·μg/ml following IV injections and 37.60 ± 6.67 h·μg/ml following IM injections. The IM-administered voriconazole exhibited a mean absolute bioavailability of 64.67 ± 11.47%.

**Discussion:**

These discoveries provide backing for the possible application of voriconazole through the intramuscular route in undulate skates and support using lower dosage regimens compared to those required for oral administration, emphasizing the importance of conducting further pharmacokinetic studies with antifungals in elasmobranchs.

## Introduction

Fungal diseases have been documented in elasmobranchs under human care in aquariums since the late 1980s. Despite their low incidence in fish, these diseases have proven to be highly lethal in various teleost and elasmobranch species (1). The involvement of several fungi, including *Purpureocilium* spp., *Exophiala* spp., and *Fusarium* spp., is frequently observed (2). Many of these fungal infections, known as invasive mycoses, are considered emerging diseases in elasmobranchs and can result in skin and eye problems or systemic spread in individuals with weakened immune systems, causing severe sickness and can ultimately lead to death (1, 3). *Fusarium solani* or *F. solani* Species Complex is emerging as a serious illness for both humans and animals and affects a wide range of elasmobranch species with Hammerhead sharks (Family *Sphyrnidae*) the most susceptible (4). Unfortunately, a lack of information about the treatment, medical protocols, and environmental management of mycoses in these species has resulted in high mortality rates (5, 6).

The treatment for these diseases in elasmobranchs should combine environmental management as well as pharmacotherapy, which is based in the use of antifungals. Antifungal drugs are commonly prescribed to improve clinical signs or lesions in affected animals, with azole antifungals one of the available therapeutic options for the treatment of systemic fungal infections in elasmobranchs (7). Azoles, a group of antifungal drugs characterized by an imidazole ring, inhibit the 14α-demethylase enzyme in fungi, disrupting ergosterol synthesis and leading to toxic metabolites that hinder fungal growth (8, 9). Therefore, a dual mechanism of action occurs: the absence of ergosterol induces a fungistatic effect and the accumulation of reactive oxygen species (ROS) resulting from the loss of membrane integrity and organelle functionality, along with toxic metabolites, leads to a fungicidal effect (10).

Although different azoles have been used empirically for the treatment of fungal diseases in elasmobranchs and other aquatic animal species, there are no studies that prove their kinetic disposition and efficacy. Among azole antifungals, voriconazole appears to be the treatment of choice, as it has produced one of the lowest minimum-inhibitory concentrations (MIC) against *Fusarium solani* during *in vitro* studies, with a MIC_90_ of 2 μg/mL. Furthermore, voriconazole has also provided very low MIC for other fungi isolated from elasmobranchs such as *Purpureocilium lilacinus* (0.5 μg/mL) (4, 11). However, further evidence regarding the use of this drugs in elasmobranchs is necessary.

The pharmacokinetic behavior of voriconazole is well-documented across a broad range of animal species, in which this drug is employed for its promising pharmacological properties, tolerability and efficacy. Dogs tolerated voriconazole administered orally at 6 mg/kg with a C_max_ of 3.07 μg/mL, while cats tolerated 4 to 6 mg/kg, resulting in a C_max_ of 2.2 μg/mL without showing adverse effects. Dogs showed a shorter t_1/2β_ (3.13 h) than cats (40.50 h), but both maintained detectable plasma concentrations 24 h post-administration (12, 13). When dealing with aquatic mammals, belugas (*Delphinapterus leucas*) treated orally with voriconazole at 3 mg/kg twice daily, reached plasma concentrations of 2 μg/mL, which were effective for the treatment of *Fusarium solani* infections in this species (14). In avian species, African penguins (*Spheniscus demersus*) given oral voriconazole at 5 mg/kg, attained a C_max_ of 1.89 μg/mL, with an extended elimination phase lasting 10.92 h (15). There seems to be great interspecific variability in voriconazole tolerance in reptiles. Deaths of cottonmouth snakes (Agkistrodon piscivorous) were reported 12 h after subcutaneous administration of 5 mg/kg. Other reptile species, including green sea turtles (*Chelonia mydas*), western pond turtles (*Actinemys Marmorata*), and red-eared sliders (*Trachemys scripta elegans*), have tolerated voriconazole well at subcutaneous and/or oral doses of 10 mg/kg (16–19). These interspecific variations underscore the limitations of interspecific drug and dosages extrapolation, while showing the importance of performing pharmacokinetic, pharmacodynamic and toxicity studies in the different animal groups and species.

Very limited information is available for azole use in fish, with a miconazole study in cyprinids (*Labeo rohita*) revealing no adverse effects after oral administration of 25.22 mg/kg, reaching a C_max_ of 20.28 μg/mL, with an extended elimination half-life of 77 h (20). When focusing on elasmobranchs, voriconazole is mainly prescribed orally at dosages ranging from 5 to 50 mg/kg every 12 h; recent studies even recommend oral dosages of voriconazole over 50 mg/kg in sharks, with therapeutic drug monitoring (6). In addition, there are empirical reports of satisfactory results after the parenteral administration of voriconazole at 4 mg/kg intramuscularly every 48 h to lesser devil rays (*Mobula hypostoma*) and 4 mg/kg intravenously at a single dose to a Bonnethead shark (*Sphyrna tiburo*) (21). The mentioned medical reports dealing with fungal infections in elasmobranchs emphasize the lack of scientific information in this field and show that the determination of species-specific voriconazole pharmacokinetics in sharks is needed. Considering that the extrapolation of pharmacotherapeutic protocols is very limited in fish, the initial step toward establishing safe and effective dosing regimens in these animals is the conduction of pharmacokinetic studies.

Recent studies have advanced in the field of pharmacokinetic research in elasmobranchs, establishing safe and efficient methodologies applicable to the study of various drugs (7, 22–24). These investigations have revealed significant interindividual variabilities and low oral absorption of certain drugs in elasmobranchs, prompting consideration of alternative parenteral administration routes such as intramuscular injection (22–25). Oral administration in elasmobranchs has proven challenging and can be voluntary or forced; voluntary administration depends on the animal to voluntarily eat the piece of food loaded with the medication and has the problem that when sharks and rays are sick, they often develop anorexia. The second option for oral administration is forced feeding via delivering whole food items using tongs or via gavage administration, which require the capture and restraint of the animals and often leads to regurgitation of the medication (7). The combination of a short esophagus and a large, expandable stomach enables elasmobranchs to store large prey or quantities of food (26). These anatomical features allow them to easily regurgitate items that they cannot digest, posing a complication when administering drugs orally and producing great interindividual variations in pharmacokinetic studies administering the medication orally (24). Detailed observation of the animal is necessary after administration to prevent voluntary regurgitation, whether associated with handling or not (7). Additionally, the difficulty of a lack of scientific data for these species is increased as previous pharmacokinetic studies have shown great interspecific differences in the pharmacokinetic properties for some drugs in this group of animals (23). This is concerning, as the efficacy of antifungal treatments is strongly related with the maintenance of optimal drug levels in plasma (8, 27). Given the difficulties associated with oral administration, together with the high oral doses required in sharks (30–50 mg/kg), parenteral administration may offer a more efficient option in elasmobranchs, allowing dosage reductions while maintaining elevated plasma concentrations. Despite being in an early stage, the possibilities for research and pharmacokinetic studies of antifungals in sharks and rays are very broad.

Voriconazole is available in oral formulations (tablets and suspension) for human use, as well as solution for intravenous administration; intravenous voriconazole, despite only being available for human use, has been also administered intramuscularly and subcutaneously in different animal species without showing adverse effects (12, 13, 15–17, 28). The application of voriconazole in elasmobranchs for clinical purposes, while currently limited, shows promise as an efficacious treatment for fungal infections. Nevertheless, there is a need for comprehensive investigations into the pharmacokinetics, pharmacodynamics, and toxicity profiles to thoroughly evaluate the viability of azoles in these unique species. This study intended to provide some light in this matter, evaluating the pharmacokinetic profile of voriconazole in elasmobranchs following administration via less frequent routes: the pharmacokinetic study of intravenous and intramuscular administrations.

## Materials and methods

This experimental study was designed as an observational, prospective, consecutive study with a washout period of 8 weeks. The processes related to the management of animals and the collection of samples were executed in conformity with and received authorization from the Animal Care and Welfare Committee at the Oceanogràfic of Valencia, as well as the Generalitat Valenciana, with the project identification codes OCE-22-19 and 2024-VSC-PEA-0091.

### Animals and experimental conditions

A group of six adult undulate skates (*Raja undulata*) (3 males and 3 females) were included in this study. These skates had been housed and cared for within the Oceanogràfic of Valencia^1^ for at least 5 years. The eligibility of these skates for participation was meticulously established through comprehensive health assessments, encompassing clinical histories, physical examinations, hematology evaluations, and plasma biochemistry analyses using analytical records from the aquarium and previously published hematology and plasma chemistry values for the species as a reference (29). These assessments confirmed their overall well-being. Upon the commencement of the study, the weights of all the skates were recorded, exhibiting a weight range spanning from 3.3 to 4.8 kg. The computed mean, accompanied by its standard deviation (± SD), for these weight measurements was documented as 4.13 ± 0.49 kg. Sexual classification was determined based on the presence or absence of claspers, while the categorization of these individuals as adults relied on their size, in accordance with established adult size ranges that had been previously documented (30, 31). Furthermore, all specimens exhibited evidence of reproductive activity, substantiated by frequent observations of male–female mating encounters and the deposition of eggs by female individuals.

During the pharmacokinetic (PK) study, the skates were temporarily relocated to 10,000-liter cylindrical tanks situated within the quarantine facility of the aquarium. The environmental conditions within these tanks were rigorously controlled, featuring: 12 h artificial light cycle followed by a 12 h darkness period; a constant water temperature maintained at 18°C; a salinity of 34 g/L in the water; pH levels maintained within the range of 7.9 to 8.1; negligible ammonia presence and a maximum nitrite and nitrate concentration of 0.05 ppm and 50 ppm, respectively. Furthermore, temporary tanks included access to shaded areas and environmental enrichment opportunities for resting. The feeding regimen consisted of providing the skates with previously thawed portions of teleosts and cephalopods once daily, six days per week, with a minimum fasting period of 24 h prior to the initiation of the study. Throughout the study, vigilant visual monitoring was conducted to promptly identify any potential clinical abnormalities associated with the handling process, blood collection procedures, or drug-related toxicity.

### Experimental protocol and sampling

This study used a single dose of 4 mg/kg, rather than the much higher dose prescribed orally (30–50 mg/kg every 12 h) in previous clinical case reports with elasmobranchs because this low dose administered intramuscularly to different ray species had already produced a decrease in the fungal load and an improvement of the clinical signs in previous reports (6, 21). In addition, a pilot study with 4 mg/kg IM voriconazole using two adult undulate skates at Oceanogràfic had already showed plasma concentrations considered clinically effective in elasmobranchs.

Voriconazole was administered at a single dose of 4 mg/kg to all six undulate skate individuals via intravenous (IV) and intramuscular (IM) route, with a washout period of eight-week between studies. For both administration routes parenteral voriconazole (Voriconazole Normon^®^ 200 mg powder for injection, Barcelona 08013, Spain) was diluted to a concentration of 20 mg/mL using sterile water for injection and administered using a 23-gauge (0.6 × 25 mm) needle attached to a 1 mL syringe. Voriconazole dosages ranged from 13.2 mg to 19.2 mg, and injection volumes ranged from 0.66 mL to 0.96 mL. Skates were carefully captured with a rubber net and manually restrained underwater.

In the case of IV administration, animals were placed in dorsal recumbency to induce tonic immobility (TI). Vascular access was achieved through the caudal vasculature using a ventral approach, and administration was slow and constant, ensuring that vascular access was maintained throughout (5).

**Figure 1:**
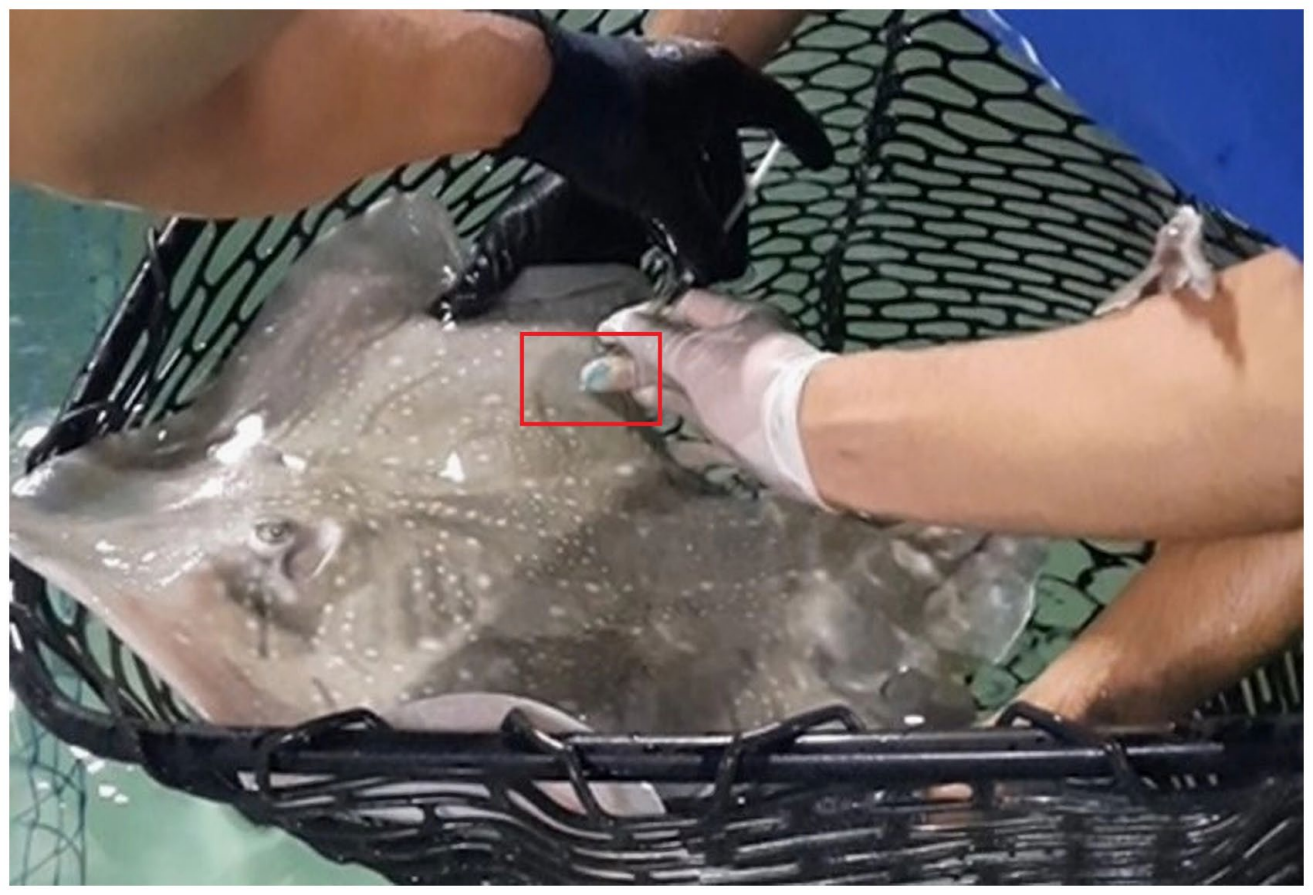
Injection site (pectoral fin musculature) utilized for the intramuscular (IM) administration of voriconazole in undulate skate (*R. undulata*) during this study (red square). Please observe that the animal is manually restrained dorsoventrally during the drug administration process. Quarantine facilities, Oceanogràfic of Valencia, Spain.

For IM administration, animals were kept in a dorsoventral position, with the injection site above water. The injection was performed dorsally, into the pectoral fin musculature (see Figure 1), with the needle inserted approximately 20 mm into the muscle. Pressure was applied at the injection site post-administration to minimize drug leakage (5).

Blood extraction utilized a ventral approach to the caudal vasculature employing a 21-gauge (0.8 × 40 mm) needle attached to a 1-ml syringe. Each skate was positioned in dorsal recumbency to induce TI, optimizing blood collection efficiency while minimizing the risk of muscle damage. The tail and posterior half of the animal were raised to the surface, while the head and gills remained submerged. Blood samples of 0.4 mL each were obtained before voriconazole administration and at specified intervals following voriconazole IV or IM administration: 15, 30 min, and 1, 1.5, 2, 4, 8, 12, 24, and 36 h. All collected samples underwent separate processing.

To ensure that dosing regimes are safe, pharmacological studies should ensure safety, both in terms of drug dosage and potential side effects, as well as possible adverse effects caused by the experimental procedure. The evaluation of skate behavior in this study was conducted by veterinarians and the responsible aquarist, who were familiar with the pretreatment behavior of the animals and visually observed the animals during and after sampling.

### Blood processing

Following blood extraction, samples were directly placed into 1 mL lithium heparin tubes (Aquisel^®^ 1 mL 12 × 55mm, AQUISEL S.L., Abrera 08630, Spain). These samples were stored at 4°C, transported to the aquarium’s laboratory, and processed within 45 min of collection. In the laboratory, heparin tubes were centrifuged at 590 g for 5 min at room temperature (24°C) using an Ortoalresa^®^ centrifuge (Ortoalresa^®^ RT106 Na 170,007/01, 132 mm rotor radius, 35-degree angle fixed, Ortoalresa-Alvarez Redondo S.A., Daganzo de Arriba 28,814, Spain). The resulting plasma was collected and transferred to 1.5 mL Eppendorf tubes, which were subsequently frozen at −20°C and dispatched to the Pharmacology and Toxicology Department of the Complutense University of Madrid for the measurement of voriconazole concentrations.

### Voriconazole quantification

Voriconazole concentration in each plasma sample was determined using a combination of previously described reverse-phase high performance liquid chromatographic methods, validated for its use in elasmobranch plasma at the laboratory of the Department of Pharmacology and Toxicology of the Complutense University of Madrid (32, 33). In this study, a C18 column (Mediterranean Sea C-18 column; Teknokroma Analítica S.A., Barcelona 08173, Spain) was mounted to the chromatography system (Hitachi High-Tech Corporation; equipped with a 5,160-model injection pump with manual purge valve, 5,280-model auto-injector with cooling unit, and a 100 μL syringe, 5,410-model variable ultraviolet detector, 08210, Barbera Del Valles, Barcelona, Spain). A mixture of Milli-Q^®^ filtered H_2_O an Acetonitrile (60:40 [vol:vol]) was used as a mobile phase, which was delivered via an isocratic flow at a rate of 0.8 mL/min. The wavelength of the UV detector was 262 nm. Chromatographic peak integration was employed for drug quantification. In drug concentration measurements, 100 μL of shark plasma were combined with 25 μL HClO_4_ (1 M). It was vortexed for 1 min, mixed with 400 μL Methanol, vortexed again for 30 s and then centrifuged at max G (14,000 rpm) for 5 min. Finally, 200 μL supernatant were collected and injected into the chromatography system. A calibration curve constructed using methanol solutions of known voriconazole concentrations (ranging from 0.05 to 15 μg/mL), exhibited linear absorbance at the studied concentrations (*R*^2^ > 0.99). The limit of quantification was 9 ng/mL, with inter- and intra-assay variability always under 6%. The mean voriconazole recovery rate in undulate skate plasma samples using this protocol resulted 80%, determined by adding known concentrations of voriconazole (Sigma-Aldrich Química SA, Tres Cantos 28,760, Madrid, Spain) to blank undulate skate plasma.

### Pharmacokinetic and statistical analysis

The number of animals used in this study was carefully selected using the GRANMO sample size calculator software (version 7.12, REGICOR, IMIM, Barcelona, Spain).

The arithmetic means of the maximum plasma concentration (C_max_) and the mean time required to attain C_max_ (T_max_) were directly computed from the plasma concentration profiles acquired during the study.

A non-compartmental analysis was executed using a commercially available software (PK Solutions, version 2.0, Summit Research Services, Montrose, United States) for the estimation of the rest of the PK parameters. The analysis encompassed the determination of several essential pharmacokinetic parameters, notably, the plasma concentration extrapolated to time zero (C_0_), the elimination half-life (t_1/2β_), the area under the plasma concentration-time curve based on observed data points (AUC_t_), the area under the plasma concentration-time curve extrapolated to infinity (AUC_inf_), and the mean residence time (MRT).

The calculation of absolute IM bioavailability (F) for voriconazole was achieved by comparing the AUC following IM and IV administration. Additionally, two key parameters, the apparent volume of distribution in pseudo-equilibrium conditions (Vd) and the systemic clearance (Cl), were estimated following IV administration. To estimate the Vd and Cl after IM administration, a correction is performed based on the calculated F for this route of administration. The mean absorption time (MAT) was determined as the difference between the MRT for IM administration and the MRT for IV administration.

This article reports plasma concentrations and pharmacokinetic parameters with their corresponding means and standard error of the mean (± SEM). A Mann–Whitney *U* test was run to evaluate statistical differences between both dosing routes, using the statistical software package SPSS Statistics (IBM SPSS Statistics for Windows, Version 25.0. IBM Corp., Armonk, NY, United States). Differences were considered statistically significant when *p* < 0.05.

## Results

The administration of voriconazole using IM and IV routes to the undulate skates was well tolerated in all animals and produced plasma concentrations over MIC for the main fungal pathogens affecting elasmobranchs (0.5–2.0 μg/mL) for periods over 12 h for both administration routes.

Voriconazole seemed to be well tolerated following the administration protocols outlined in this study, as all animals exhibited normal levels of activity, appetite, reproductive activity and swimming behavior. No abnormal clinical signs were observed in the animals throughout the studies nor 8 weeks after the conclusion of each study.

**Figure 2:**
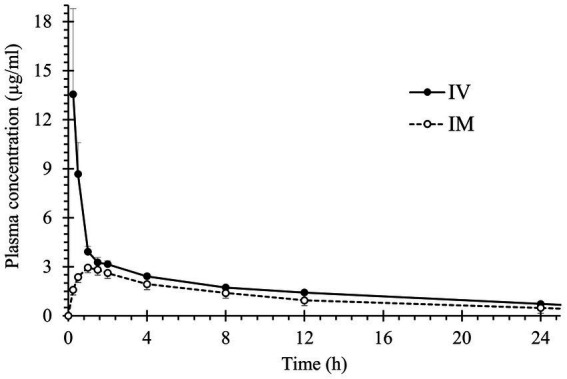
Mean ± SEM plasma concentrations of voriconazole in undulate skates (*R. undulata*; *n* = 6) after administration of a single IV (solid circles, continued line) or IM dose (open circles, discontinued line) (4 mg/kg).

Table 1 provides a summary of the pharmacokinetic parameter estimates for voriconazole administration at a dose of 4 mg/kg, both intravenously (IV) and intramuscularly (IM). The average plasma concentrations following IV and IM administration of voriconazole to *R. undulata* are depicted in Figure 2.

**Table 1:** Pharmacokinetic parameters of voriconazole administered at a single dose of 4 mg/kg IV or IM in undulate skates (*R. undulata*; *n* = 6) maintained under human care.

		IV administration (*n* = 6)	IM administration (*n* = 6)			
Parameter	Unit	Mean	SEM	MEAN	SEM	*p*
C_0_	μg/ml	27.19	7.15	–	–	–
T_max_	h	–	–	1.33	0.17	–
C_max_	μg/ml	–	–	2.98	0.28	–
t_½β_	h	11.18	1.32	9.59	1.38	0.485
AUCt*	h∙μg/ml	47.46	3.39	27.92	5.06	0.026
AUCinf*	h∙μg/ml	58.14	2.79	37.60	6.67	0.041
MRT	h	13.17	2.09	14.43	2.19	1.000
Vd	L/kg	1.12	0.14	0.98	0.10	0.485
Cl	ml/h/kg	69.2	3.0	78.6	11.9	0.394
F	%	–	–	64.67	11.47	–
MAT	h	–	–	4.56	1.03	–

T_max_, time required to achieve maximum plasma concentration; C_0_, extrapolated concentration at 0 h after IV administration; C_max_, maximum plasma concentration; t_1/2_ β, elimination half-life; AUC_t_, area under the plasma concentration curve computed using observed data points only; AUC_inf_, area under the curve extrapolated to infinity; MRT, mean residence time; Vd, distribution volume in pseudo-equilibrium conditions; Cl, clearance; F, bioavailability; MAT, mean absorption time. * Indicates the presence of statistically significant differences (*p* < 0.05; Mann–Whitney U test) between IV and IM administration protocols.

**Figure 3:**
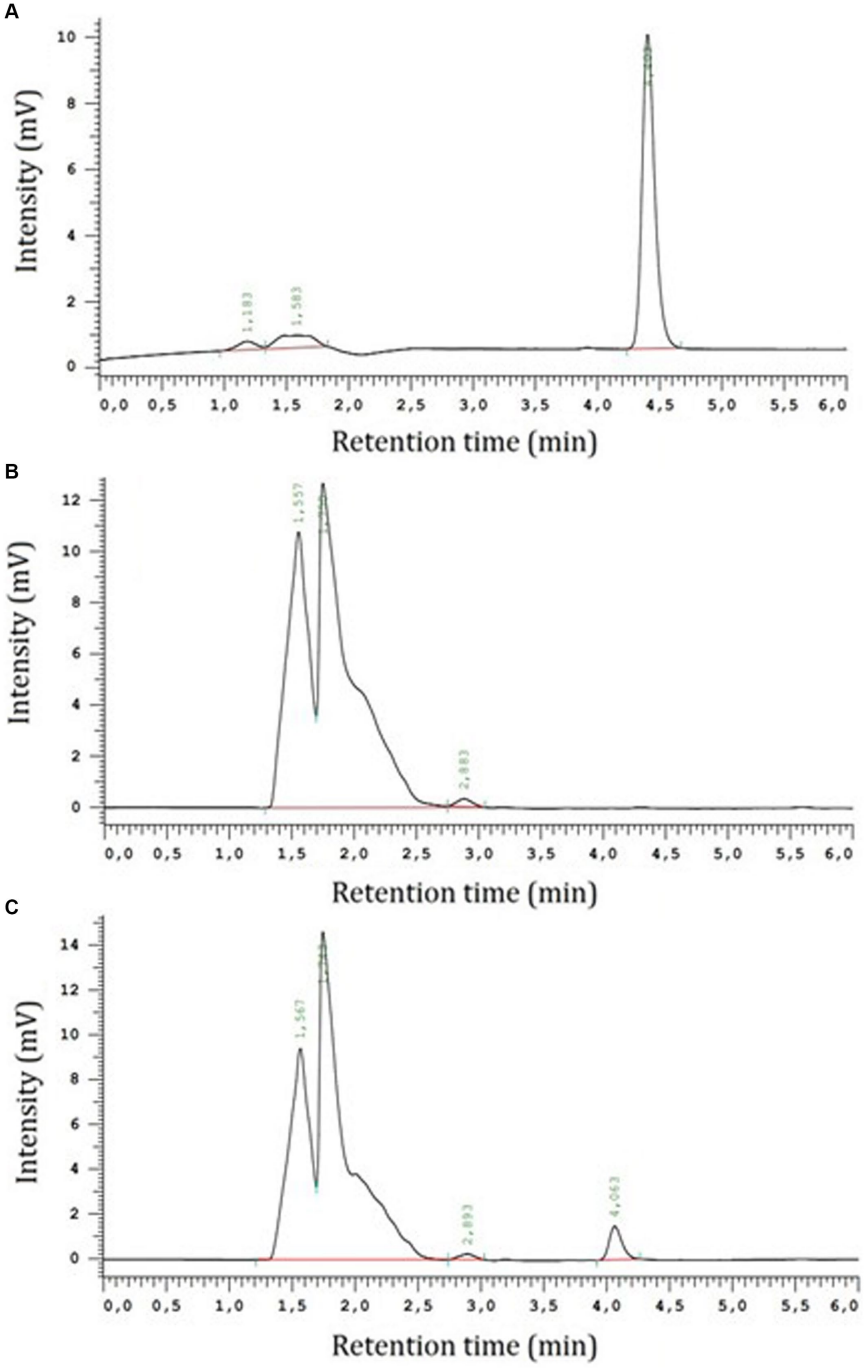
Representative chromatograms of voriconazole (Sigma-Aldrich Química SA., Tres Cantos 28,760, Madrid, Spain) at 5 μg/mL concentration in methanol, retention time was 4.40 min and AUC was 67,484 AUFS·min^−1^ **(A)**; blank undulate skate (*R. undulata*) after processing using the methodology described by Blanco Dorado et al. (32) and Zhang et al. (33) **(B)**, plasma and a sample containing voriconazole at 5 μg/mL concentration in undulate skate plasma after processing using the methodology described by Blanco Dorado et al. (32) and Zhang et al. (33); retention time was 4.06 min and AUC was 10,474 AUFS·min^−1^ **(C)**. Hitachi High-Tech Corporation, 08210, Barbera Del Valles, Barcelona.

Voriconazole showed a rapid absorption after IM administration, with a T_max_ of 1.33 ± 0.37 h, and a MAT of 4.56 ± 1.03 h. Bioavailability for IM administration was 64.67 ± 11.47%. No statistical differences were detected for drug distribution, with a Vd of 1.12 ± 0.14 L/kg for IV and 0.98 ± 0.10 L/kg for IM administration (*p* = 0.485; Mann–Whitney *U* Test); these values are close to 1 L/kg and suggest that the drug has a good distribution across the entire organism, without apparent accumulation in any tissue. Elimination was slow and progressive after both IV and IM administrations, with similar rates for both dosing routes (t_1/2β_ = 11.18 ± 1.32 and 9.59 ± 1.38 h, respectively; and MRT = 13.17 ± 2.09 and 14.43 ± 2.19 h, respectively). Furthermore, after incorporating the value of bioavailability into the systemic clearance calculation following IM administration, the values of this parameter did not differ statistically from those estimated for the IV route (*p* = 0.394; Mann–Whitney *U* Test), resulting 69.2 ± 3.0 mL/h/kg for IV and 78.6 ± 11.9 mL/h/kg for IM administration (Figure 3).

## Discussion

When it comes to selecting the most appropriate active compound and the dosing regime, veterinary clinicians working with elasmobranchs still face the challenge of lacking reliable and accurate scientific data in the field of pharmacology and pharmacotherapeutics for most of the drugs prescribed in sharks and rays (6, 7). To the authors knowledge, this is the first pharmacokinetic study with voriconazole in fish. The promising pharmacokinetic results obtained in this study, together with the great interspecific drug kinetic differences observed for other medications in teleost and elasmobranchs, even for species with similar biological and ecological characteristics, justify the development of further pharmacokinetic studies with voriconazole in different fish species (23).

As previously mentioned, azole antifungals frequently represent the primary choice for both preventing and treating invasive fungal infections; they are frequently administered for extended durations, spanning from weeks to months. The prolonged use of azoles is linked to adverse effects such as hepatotoxicity, hypokalemia, hyponatremia, hormone-related impacts, and rarely, adrenal insufficiency (34). Given that elevated plasma levels of voriconazole have been associated with an elevation in hepatic enzymes and hepatotoxicity, trough concentrations of voriconazole should be monitored not only to ensure successful antifungal therapy but also to avoid hepatic damage (35). Access to pharmacokinetic testing can be limited in veterinary medicine and especially in the clinical management of elasmobranchs. Having access to pharmacokinetic data in multiple different species can assist in conservatively developing the best regimen for a given species, recognizing that interspecies differences can be significant (7, 36).

A limitation to our study was that in an effort to decrease the total blood volume to be collected from the animals during the pharmacokinetic study (only 0.4 mL per sample), no complete blood analysis was performed. Further studies evaluating the pharmacokinetics of voriconazole in elasmobranchs should include routine blood tests (eg. hematology, plasma chemistry, plasma protein electrophoresis) to assess for subclinical impacts on organ function and animal health. It is important to note that blood analyses in a single dose study such as the one performed, while offering valuable insights into potential adverse effects of concurrently administered drugs and aiding in the evaluation of acute toxicity alongside pharmacokinetic studies, do not comprehensively evaluate the impact of voriconazole on hepatic function (34, 35, 37).

Despite the great interindividual and interstudy differences observed with oral drug administration in sharks and rays, oral dosing is still the most common administration route for voriconazole in elasmobranchs (21). Results provided by this study are relevant for aquatic animal veterinarians and researchers because they present novel data on the PK properties of voriconazole in elasmobranchs, evaluating two less frequent administration routes: intravenous and intramuscular administration. This study suggests that the selection of an intramuscular administration route could allow a significant reduction in the dosages of voriconazole needed to achieve effective plasma concentrations when compared to the posology regimes currently recommended for elasmobranchs (6). The relatively elevated plasma concentrations after the IM administration of 4 mg/kg in the undulate skate (2.98 μg/mL) when compared to those obtained after the oral administration of 50 mg/kg every 12 h in the scalloped hammerhead shark (1.2 μg/mL) suggest that oral bioavailability of voriconazole in elasmobranchs could be limited. This phenomenon has been previously described in elasmobranchs following oral administration of other drugs and active compounds. The cause of the great interindividual variation and low plasma concentrations of orally administered compounds in elasmobranchs requires further study (22). This is important as oral administration is the most frequently used dosing route, because it allows treatment but avoids capture, handling and the potential stress response or capture related injuries (38). Treating diseases elasmobranchs intramuscularly instead of orally, would decrease the possibility of under- or overdosing due to the strong interindividual variations observed. Another important factor that may contribute to limited oral bioavailability of different drugs in elasmobranchs, and would be avoided with intramuscular administration, is pre-systemic elimination or hepatic first-pass phenomenon (39). Studies that quantify drug metabolites in plasma and tissues are needed to determine if hepatic first-pass effect plays a role in interindividual and interspecific differences in drug plasma concentrations in elasmobranchs. Our results reveal that voriconazole is rapidly absorbed in the undulate skates after IM administration, with a T_max_ of 1.33 ± 0.17 h and a MAT of 4.56 ± 1.03 h, leading to high plasma concentrations (Cmax of 2.98 ± 0.28 μg/mL). Elimination seems to be more prolonged in time than absorption, with a Cl of 78.6 ± 11.9 mL/h/kg. These auspicious PK results could support the prescription of multiple-dose treatment protocols, in which voriconazole could be administered every 12 h under the studied conditions. Although our results are promising the following factors should be considered with regard to the intramuscular administration of voriconazole in this study.

Drug leakage following intramuscular administration of medications in elasmobranchs is a known issue and must be considered in this study, though manual pressure was applied and no leakage was appreciated. This could have reduced the bioavailability and drug plasma concentrations, as previous studies have reported in teleost and elasmobranch fish (7, 22). Future research goals include quantifying the amount of drug expelled from the injection site and exploring strategies to minimize leakage if detected, such as sealing the injection site, using higher concentration formulations or distributing the dose across multiple injection sites. However, it should be noted that the main goal of this study was to evaluate the PK of voriconazole replicating the current administration protocols for voriconazole in elasmobranch clinical management.

Another pharmacological phenomenon which has been described in fish and which could be also reducing drug bioavailability after parenteral administration in elasmobranchs is the renal portal system, which collects blood from the caudal fin, which then passes through the kidneys before returning to the heart; this can increase nephrotoxicity or pre-systemic renal excretion and modify their distribution (23, 39). Given that the recommended site for intravenous administration in elasmobranchs is the caudal vessels and for intramuscular administration it is the epaxial musculature lateral to the dorsal fin (also in the caudal half of the animal), this administration may be affected by the renal portal system, and its effect must be taken into account in further studies. Future research goals include the studying the PK differences between the administration in the cranial and caudal halves of sharks and rays. Therefore, future trials would be necessary to rule out a possible interaction of the renal portal system in the distribution and elimination of certain drugs such as voriconazole in this group of animals.

Clinicians should note that in the context of fungi, a robust correlation between *in vitro* minimum inhibitory concentration (MIC) levels and effective *in vivo* drug concentrations is often elusive (6, 27, 35). This discrepancy can be attributed to various factors influencing the complex dynamics of drug interactions within a living organism, making it challenging to predict the *in vivo* effectiveness based solely on *in vitro* MIC values (8). Consequently, comprehensive, and context-specific assessments are essential to better understand and optimize the therapeutic outcomes of antifungal treatments.

Former studies with voriconazole have shown that metabolism can significantly influence the kinetic behavior, revealing a substantial reliance on the functionality of certain cytochrome P450 enzymes, specifically 2C19, 2C9, and 3A4 (40). Earlier studies emphasize the need to carefully explore how drugs are processed by CYP enzymes, cautioning against assuming uniform reactions across various animals. Fish, in particular, exhibit unique responses compared to mammals in this aspect (41). The role of this enzymatic system in elasmobranchs and its influence on drug elimination is currently unknown, but it could serve as a rationale for explaining the substantial kinetic differences observed following oral administration of various drugs in this group of animals. The hepatic characteristics also exhibit great interspecific variability, with elasmobranchs manifesting a liver constituting a substantial proportion, up to 23% of their total body weight, primarily comprised of lipids. In addition, elasmobranchs lack cavitary adipose tissue and opt for hepatocytes as the primary site for lipid storage (42). This distinctive hepatocellular lipid storage, in conjunction with the extent of liver cell exposure and lipid concentration, introduces considerable nuances in drug pharmacology within elasmobranchs (35, 43). The likelihood of a progressive decline in plasma concentrations to suboptimal drug levels during prolonged treatment necessitates systematic monitoring to ascertain and address the implications of enzymatic induction on the drug’s metabolism.

Furthermore, it should be considered that the clinical management of fungal diseases in this group of animals must have a multimodal approach (3–6). First, environmental considerations should be implemented to slow down the advancement of the disease and attempt to cure the animals; these environmental interventions include an adjustment of salinity while still maintaining a range compatible with the species survival; temperature regulation; and an increase in oxygen saturation (5). The clinician working with teleost and elasmobranchs should be conscious that variations in water temperature and salinity can produce differences in drug absorption, distribution, metabolization and excretion (7, 44). Given that temperature regulation is a key consideration when treating fungal diseases in fish and that variations in environmental temperature can produce differences in drug PK parameters, further studies should evaluate how temperature affects voriconazole absorption, distribution and elimination in the different elasmobranch species, as this could be key to achieve clinically effective concentrations in plasma and tissues.

Food supplementation with vitamins and immunostimulants is also frequently considered to enhance the animal’s immunity when dealing with fungal diseases in elasmobranchs (15). Clinicians should consider that this supplementation together with the use of concomitant medication, such as antimicrobials to treat secondary infections, can lead to pharmacological interactions and also have an influence on antifungal pharmacokinetics (8, 27). Future research goals include evaluating the effect of feeding on the kinetics of orally administered voriconazole, given the unique characteristics of their digestive system and the importance of this route of administration in these animals.

As mentioned throughout this article, voriconazole has provided promising pharmacodynamic data and tolerability in different aquatic animals including elasmobranchs (4, 11, 45). This, together with the fact that azole antifungals have proven great efficacy in the pharmacotherapeutic treatment of mycotic infections in fish, justified the selection of voriconazole to be evaluated in our study (4, 5, 46). The results provided by our study suggest that voriconazole could be a promising antifungal drug to be used intramuscularly in elasmobranchs due to its good kinetic properties and tolerance. The data provided in this study provides the first pharmacokinetic basis for intramuscular and intravenous dosing of voriconazole in elasmobranchs, and continues to build the foundation of scientifically based pharmacotherapeutics in this taxonomic group. Results obtained from this study also highlight the important differences in drug disposition among administration routes in this group of animals and underscores the need for future pharmacokinetic and pharmacodynamic studies evaluating the different drug delivery methods in sharks and rays.

## Conclusion

Voriconazole administered intravenously and intramuscularly to undulate skates at a dose of 4 mg/kg achieved higher plasma concentrations compared to those reached in other elasmobranch species after the administration of the same drug at dosages up to 50 mg/kg via oral route, while showing no apparent adverse effects. Both IV and IM routes of administration allowed for plasma levels of voriconazole over the *in vitro* calculated MICs for *Fusarium solani* and *Purpureocilium lilacinus* in elasmobranchs for at least 12 h. These results support the use of voriconazole via parenteral administration in undulate skates and justify the study of intramuscular administration in other shark and ray species. Further pharmacodynamic and toxicologic studies should be performed with voriconazole in other elasmobranch species to ensure the efficacy and safety of this drug and the proposed dosing regime.

## Data availability statement

The raw data supporting the conclusions of this article will be made available by the authors, without undue reservation.

## Ethics statement

The animal study was approved by Animal Care and Welfare Committee at the Oceanogràfic of Valencia. Project identification code OCE-22-19. The study was conducted in accordance with the local legislation and institutional requirements.

## Author contributions

DC-C: Conceptualization, Investigation, Methodology, Writing – original draft, Writing – review & editing. CR-S: Methodology, Writing – review & editing. SR-L: Data curation, Formal analysis, Investigation, Writing – review & editing. DG-P: Funding acquisition, Project administration, Resources, Writing – review & editing. TE: Conceptualization, Data curation, Methodology, Supervision, Writing – review & editing. PM-E: Conceptualization, Data curation, Formal analysis, Investigation, Methodology, Resources, Software, Supervision, Writing – original draft, Writing – review & editing.

## Footnotes

^1^http://www.cac.es/oceanografic
